# Effects of Cryptorchidism on the Semen Quality of Giant Pandas from the Perspective of Seminal Plasma Proteomics

**DOI:** 10.3390/genes15101288

**Published:** 2024-09-30

**Authors:** Yicheng Qian, Yuliang Liu, Tao Wang, Shenfei Wang, Jiasong Chen, Feiping Li, Mengshi Zhang, Xianbiao Hu, Juan Wang, Yan Li, Ayala James, Rong Hou, Kailai Cai

**Affiliations:** 1 Antibiotics Research and Re-Evaluation Key Laboratory of Sichuan Province, Sichuan Industrial Institute of Antibiotics, School of Pharmacy, Chengdu University, Chengdu 610106, China; qianyicheng@stu.cdu.edu (Y.Q.); wangtao3@cdu.edu.cn (T.W.); 2 Chengdu Research Base of Giant Panda Breeding, Chengdu 610081, China; sdliuyuliang@163.com (Y.L.); wangsf6688@126.com (S.W.); chenjs91@163.com (J.C.); lifeiping822@163.com (F.L.); zms2233@126.com (M.Z.); xtt671384@163.com (X.H.); ccwang@panda.org.cn (J.W.); liyan@panda.org.cn (Y.L.); ayalajames@msn.com (A.J.); hourong2000@panda.org.cn (R.H.); 3 Sichuan Key Laboratory of Conservation Biology for Endangered Wildlife, Chengdu 610081, China

**Keywords:** giant panda, seminal plasma, proteomics, cryptorchidism

## Abstract

Giant pandas are an endangered species with low reproductive rates. Cryptorchidism, which can negatively affect reproduction, is also often found in pandas. Seminal plasma plays a crucial role in sperm–environment interactions, and its properties are closely linked to conception potential in both natural and assisted reproduction. The research sought to identify seminal fluid protein content variations between normal and cryptorchid giant pandas. **Methods:** Using a label-free MS-based method, the semen proteomes of one panda with cryptorchidism and three normal pandas were studied, and the identified proteins were compared and functionally analyzed. **Results:** Mass spectrometry identified 2059 seminal plasma proteins, with 361 differentially expressed proteins (DEPs). Gene ontology (GO) analysis revealed that these DEPs are mainly involved in the phosphate-containing compound metabolic, hydrolase activity, and kinase activity areas (*p* ≤ 0.05). The KEGG functional enrichment analysis revealed that the top 20 pathways were notably concentrated in the adipocyte lipolysis and insulin metabolism pathway, with a significance level of *p* ≤ 0.05. Further analysis through a protein–protein interaction (PPI) network identified nine key proteins that may play crucial roles, including D2GXH8 (hexokinase Fragment), D2HSQ6 (protein tyrosine phosphatase), and G1LHZ6 (Calmodulin 2). **Conclusions:** We suspect that the high abundance of D2HSQ6 in cryptorchid individuals is associated with metabolic pathways, especially the insulin signal pathway, as a typical proteomic feature related to its pathological features. These findings offer insight into the ex situ breeding conditions of this threatened species.

## 1. Introduction

The giant panda (*Ailuropoda melanoleuca*) is an endemic species that is uniquely cherished and recognized as a Chinese national treasure. It is a conservation-dependent threatened species, characterized by low numbers, isolated populations, and a limited reproductive rate, especially in captive populations. Female giant pandas, whether living in the wild or in captivity, experience estrus for a brief period annually; however, in captivity, fewer than 10 percent of male giant pandas are capable of naturally mating, and as a result, fewer than 30 percent of females conceive naturally [1]. Testicular descent in giant pandas generally occurs at between three and four years of age, and males reach sexual maturity at roughly eight years old. From when the giant panda testes begin to decline to the sexual maturity stage, there is an observed expansion in the size of the testes, a rise in androgen levels, and an enhancement in sperm output with each subsequent breeding season [2]. Artificial insemination (AI) is the most commonly used technique in giant panda captive breeding programs. Previous studies on the reproductive challenges of male giant pandas have focused on reproductive behaviors [3,4,5]; however, there is a relative lack of research on the quality of the semen of giant pandas and its effect on artificial insemination.

Seminal plasma is a complex biological liquid, composed of a mixture of secretions originating from the testes, auxiliary reproductive glands, and epididymis. It envelops the sperm during ejaculation and is crucial in the reproductive biology of all male vertebrate animals [6]. The seminal plasma has complex properties. The main protein components of the seminal vesicle fluid are semenogelin I but also semenogelin II, involved in the gelation (coagulation) of the ejaculatory injection, which is reflected in the inhibition of sperm movement and antibacterial activity. Prostate secretions, which account for only 20-30% of the seminal plasma volume, are in direct contact with most sperm and are the first part of the seminal plasma to face the cervical canal [7]. As well, most peptides (albeit most of them are either fragment products of SP proteins or sperm-associated peptide hormones [8]) and other enzyme components, such as glycosidases, lipocalin-type prostaglandin D2 synthase, lipase [9], or matrix metalloproteinases (MMPs), make contact with the sperm surface. These results indicate that seminal plasma is closely related to male fertility and semen quality [10,11]. The metabolic alterations triggered by interactions with seminal plasma, the attachment of its proteins to the sperm cell’s exterior, and the restructuring of the sperm membrane may all have an impact on sperm quality, the maturation process, the acquisition of motility, the process of capacitation, and the acrosome reaction [12,13,14,15,16]. Successful fertilization is contingent upon a combination of intrinsic sperm development factors and extrinsic influences from the seminal plasma. These external factors can influence sperm motility and the formation of the zygote within the female reproductive tract, thereby significantly contributing to the reproductive process [17,18]. Additionally, the seminal plasma is capable of initiating both inflammatory and immune responses [19] and can subsequently protect spermatozoa against oxidative stress [20,21]. The seminal plasma, functioning as a conduit for sperm to engage with external factors, has attributes that are closely correlated with the capacity for reproduction [22,23].

The seminal plasma proteomes of an increasing number of vertebrate species are being studied using protein separation methods combined with protein identification through mass spectrometry. In recent years, proteomic research across mammalian and poultry species has uncovered several seminal plasma proteins that are pivotal to the assessment of semen quality. For example, elevated levels of porcine seminal protein-I (PSP-I) and cathepsin B (CTSB) in boar seminal plasma have been linked to a reduction in overall and progressive sperm motility [24]. Zinc-α-2-glycoprotein (ZAG) has been found to interact with the spermatozoa surface and modulate sperm motility via the cAMP/PKA signaling pathway, as per recent studies [25]. And more studies have shown that ZAG activity is found in many species. For example, the activity of ZAG has been demonstrated in rodents to be mediated by the β3-adrenergic receptor, the upregulated cAMP pathway [26,27,28]. In addition, it is also known that ZAG is a special factor related to human sperm motility, through the activation of the cAMP/PKA signaling pathway, and thus to sperm motility regulation [29]. In rabbits, the percentage of sperm with intact membranes was correlated with the presence of seminal plasma proteins such as the FAM115 complex and tropomyosin [30]. Furthermore, seminal plasma proteins such as adhesion G-protein coupled receptor G2 (ADGRG2) and serine peptidase inhibitor Kazal-type 2 (SPINK2) are instrumental in preserving sperm motility in indigenous chicken [31].

Cryptorchidism, also known as undescended testis or maldescendus testis, is a condition that is not uncommon in many mammalian species and has been observed in black bears [32], cats [33], dogs [34], etc. It involves the failure of one or both testes to descend into the bottom of the scrotum [35,36]; instead, they may be found at a location along the normal route of testicular descent, such as in intra-abdominal, inguinal, suprascrotal, or high-scrotal positions. Congenital cryptorchidism is a prevalent abnormality in males [37], and it is linked to a higher likelihood of decreased semen quality and an increased incidence of testicular germ cell tumors (TGCT) in the future [38]. Research indicates that testicular hormones, androgens, and insulin-like peptide 3 (INSL3) play a crucial role in the descent of the testes from the intra-abdominal position into the scrotum during development. INSL3, produced by Leydig cells, is considered a sensitive marker of Leydig cell function and differentiation [39,40,41,42]. It is highly expressed in the fetal testis, downregulated after birth, and then upregulated again during puberty [43]. Apart from its role in testicular descent and cryptorchidism, INSL 3 may play important yet undetermined endocrine and paracrine roles in adults, and the absence of this hormone may indicate hypogonadism [41].

Given this phenomenon, studying the semen of giant pandas and understanding the seminal plasma quality to some extent plays an important role in the biological principles of giant panda evolution, genetic diversity, immunity, reproduction, and development. Previous research has found that the accumulation of the mouse Uchl1 protein is involved in the heat stress-induced spermatocyte apoptosis in cryptorchidism through a new pathway related to Jab1 and p27kip1 [44]. P. Krzeminska et al. (2020) analyzed the expression of four genes involved in testicular steroidogenesis (CYP17A1, CYP19A1, HSD3B2, and HSD17B3) in the scrotal testes from inguinal, unilateral cryptorchid dogs and the scrotal gonads of normal males [45].

A comprehensive proteomic study of giant panda seminal plasma was carried out to study the protein composition of seminal plasma and clarify the biological events of seminal plasma development. These results will help to clarify the regulatory role of seminal plasma proteins in sperm maturation, motility, and fertilization in the species. Following a comparison of the protein differences between the cryptorchidism group and the normal group, it is reasonable to speculate that the differences in some genes or proteins may affect the quantity and quality of the sperm of cryptorchid giant pandas.

## 2. Materials and Methods

### 2.1. Sample Collection and Preparation

For the present study, a total of four healthy male giant pandas, an 11-year-old panda (D), a 9-year-old panda (C), a 16-year-old panda (X), and a 13-year-old panda (A), were used as experimental subjects. Under normal husbandry conditions, these giant pandas were housed at the Chengdu research base of Giant Panda Breeding (simply Chengdu Panda Base) in Chengdu, China. Semen samples were collected from all four males following routine semen collection protocols by the veterinary and reproductive technology team at the Chengdu Panda Base. The giant pandas must fast and abstain from water for 12h before sampling anesthesia to clear gastrointestinal contents. As previously reported [46], sedation was performed with Ketamine (4–8 mg/kg, IM) followed by Halothane anesthesia during semen collection. The method of semen collection is electroejaculation; the instrument used is an electroejaculation apparatus (Boring, OR, USA), the voltage is set to 2–8 V, and continuous stimulation occurs for 2 s. Stimulation stops as soon as ejaculation occurs. The collected semen is collected into the semen collection cup. After resting for 30 s, the above operation was repeated, and semen was collected once for each giant panda for research [47]. All semen samples were centrifuged to obtain the seminal plasma supernatant and were stored in a freezer at −80 °C.

D is a bilateral cryptorchid with azoospermic semen. The remaining three giant pandas had normal testicle development, and their semen was identified as having normal quantity and vitality. The samples were then divided into two groups based on their two primary characteristics: the cryptorchid group (marked as AZ; contained D) and the normal group (marked as NZ; contained C, X, and A).

### 2.2. Total Protein Extraction, Quality Control, and Trypsin Treatment

The seminal plasma samples were placed in an ultrafiltration tube and centrifuged at 14,000× *g* for 15 min. Once the permeate was removed, the ultrafiltration sample was poured into a 1.5 mL centrifuge tube and adjusted with DB buffer, which contains 8 M Urea and 100 mM TEAB at a pH of 8.5. The proteins solution was treated with 10 mM DTT for 60 min at a temperature of 56 °C to achieve reduction. Afterward, the proteins were alkylated using an adequate amount of IAM for 60 min at an ambient temperature in a dark environment. The protein concentration was determined using a BCA protein assay as per the instructions of the Bradford protein quantitative kit (Beyotime, Shanghai, China), with a gradient concentration ranging from 0 to 0.5 g/L. BCA standard protein solutions and sample solutions with different dilution multiples were added into 96-well plates to fill up the volume to 20 μL, respectively. Each gradient was repeated three times. The plate was then quickly added with a 180 μL G250 dye solution and placed at room temperature for 5 min, after which the absorbance at 595 nm was measured. The standard curve was plotted using the absorbance of the standard protein solution, and the protein concentration of the sample was calculated. Furthermore, 20 μg of the protein sample was loaded onto a 12% SDS-PAGE gel for electrophoresis, with a concentrated gel run at 80 V for 20 min and a separation gel run at 120 V for 90 min. The gel was stained with Coomassie brilliant blue R-250 and decolorized until the bands were visible.

Each protein sample was adjusted to a volume of 100 μL with DB lysis buffer (8 M Urea, 100 mM TEAB, pH 8.5), followed by the addition of trypsin and 100 mM TEAB buffer. The sample was then mixed and digested at 37 °C for 4 h. Subsequently, trypsin and CaCl_2_ were added for overnight digestion. The digested sample was mixed with formic acid and the pH was adjusted to below 3, after which it was centrifuged at 12,000× *g* for 5 min at room temperature. The resulting supernatant was carefully loaded into the C18 desalting column, washed three times with washing buffer (0.1% formic acid, 3% acetonitrile), and then eluted with elution buffer (0.1% formic acid, 70% acetonitrile). The eluents from each sample were collected and lyophilized.

### 2.3. LC-MS/MS Analysis

Solution A (100% water, 0.1% formic acid) and solution B (80% acetonitrile, 0.1% formic acid) were prepared. The lyophilized powder was dissolved in 10 μL of solution A, centrifuged at 14,000× *g* for 20 min at 4 °C, and 1 μg of the supernatant was injected into a custom-made C18 Nano-Trap column (4.5 cm × 75 μm, 3 μm). Peptides were separated in a custom-made analytical column (15 cm × 150 μm, 1.9 μm). The separated peptides were analyzed by a Q ExactiveTM HF-X mass spectrometer (Thermo Fisher, Waltham, MA, USA), with an ion source of Nanospray Flex™ (ESI), a spray voltage of 2.1 kV, and an ion transport capillary temperature of 320 °C. The full scan range was from *m*/*z* 350 to 1500 with a resolution of 60,000 (at *m*/*z* 200), an automatic gain control (AGC) target value of 3 × 10^6^, and a maximum ion injection time of 20 ms. The top 40 precursors of the highest abundance in the full scan were selected and fragmented by higher energy collisional dissociation (HCD) and were analyzed in MS/MS, where the resolution was 15,000 (at *m*/*z* 200), the automatic gain control (AGC) target value was 1 × 10^5^, the maximum ion injection time was 45 ms, a normalized collision energy was set at 27%, the intensity threshold was 2.2 × 10^4^, and the dynamic exclusion parameter was set at 20 s. The raw data of MS detection were named “.raw”.

### 2.4. The Identification and Quantitation of Protein

The database search software Proteome Discoverer 2.2 (PD 2.2, Thermo) was used to search all the result spectra. The search parameters were set as follows: the mass tolerance for the precursor ion was 10 ppm and the mass tolerance for the product ion was 0.02 Da. A maximum of 2 missed cleavage sites were allowed. To improve the quality of the analysis results, the software PD 2.2 further filtered the retrieval results: Peptide Spectrum Matches (PSMs) with a credibility of more than 99% were identified as PSMs. The identified protein contained at least 1 unique peptide. The identified PSMs and proteins were retained and performed with FDR no more than 1.0%. A *t*-test was used to statistically analyze the protein quantitation results. The proteins whose quantitation was significantly different between the experimental and control groups (this project selects upregulated proteins when FC ≥ 2.0 and *p*-value ≤ 0.05 and downregulated proteins when FC ≤ 0.50 and *p*-value ≤ 0.05) were defined as differentially expressed proteins (DEPs).

### 2.5. The Functional Analysis of Protein and DEP

Gene ontology (GO) and InterPro (IPR) functional analysis were performed using the interproscan program against the non-redundant protein database (including Pfam, PRINTS, ProDom, SMART, ProSite, PANTHER) [48]. The databases of Clusters of Orthologous Groups (COG) and Kyoto Encyclopedia of Genes and Genomes (KEGG) were utilized to analyze the protein family and pathway. DPEs were employed for the Volcanic map analysis, cluster heat map analysis, and enrichment analysis of GO, IPR, and KEGG [49]. The likely protein–protein interactions were predicted using the STRING-db server [50] (http://string.embl.de/, accessed on 25 January 2024).

## 3. Results

### 3.1. Seminal Plasma Proteome Profiles of the Giant Panda and One with Cryptorchidism

**Figure 1:**
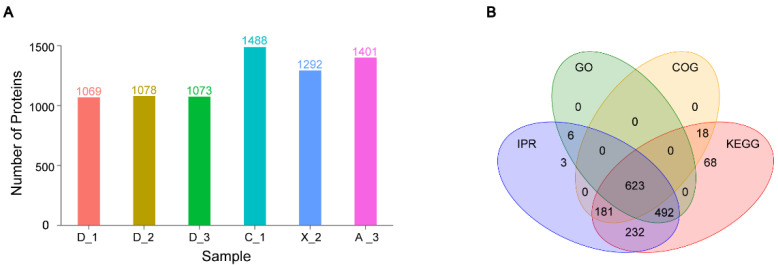
Statistics of the identified seminal plasma proteins in the giant panda. (**A**) Sample grouping statistics from tandem mass spectrometry and protein identification. (**B**) Functional annotations of proteins in public databases.

Following extraction and enzymatic digestion, the peptides derived from seminal plasma underwent analysis via liquid chromatography–tandem mass spectrometry (LC-MS/MS). This process yielded 16,785 distinct peptides, which were matched to a total of 2059 distinct proteins (Supplementary Table S1). The cryptorchid group consists of three samples, D_1, D_2, and D_3, all obtained from giant panda D, and these were utilized as repeated samples. The other three samples—C_1, X_2, and A_3—in the normal testis group were collected from giant pandas C, X, and A (Figure 1A). Among them, the average numbers of proteins identified in the cryptorchid group and normal group were 1073 and 1393, respectively.

To evaluate the proteome map of giant panda sperm and seminal plasma, we annotated proteins based on four general functional databases: GO, KEGG, COG, and IPR. The Venn diagram below depicts the four functional databases (Figure 1B). The four databases collectively annotated 623 proteins, with only a small number of proteins being individually annotated by each database. In contrast, the KEGG functional database presents a more comprehensive and extensive supply of protein.

### 3.2. Annotation and Analysis of Seminal Plasma Protein

**Figure 2:**
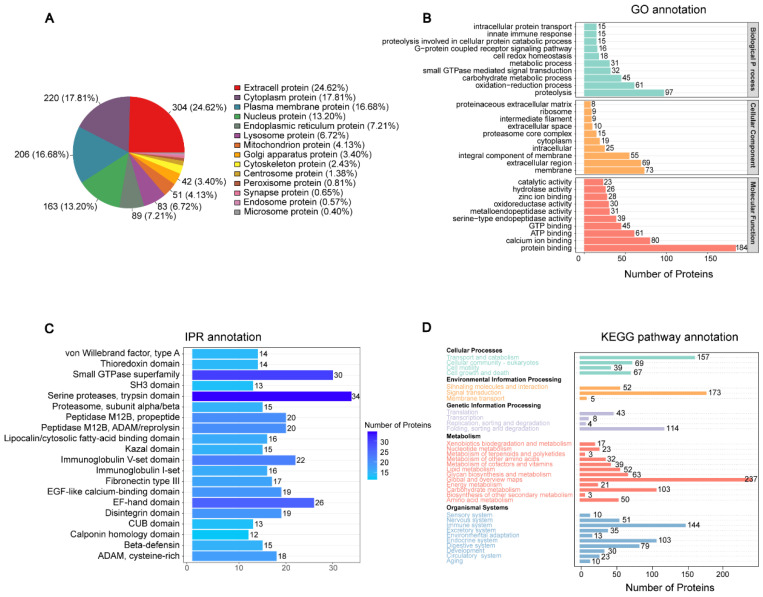
Functional annotation of the identified seminal plasma proteins in giant panda semen. (**A**) Subcellular localization of the identified proteins. (**B**) GO enrichment analysis of the identified proteins. (**C**) IPR analysis of the identified protein. (**D**) KEGG analysis of the identified proteins.

The findings reveal that the proteins were distributed across numerous subcellular compartments. The majority were found in the extracellular space, with 304 proteins (comprising 24.62%), followed by the cytoplasm with 220 proteins (17.81%), the plasma membrane with 206 proteins (16.68%), the nucleus with 163 proteins (13.20%), the endoplasmic reticulum with 89 proteins (7.21%), lysosomes with 83 proteins (6.72%), and mitochondria with 51 proteins (4.13%) (Figure 2A). The GO annotation indicates that a significant enrichment in the identified proteins was observed in molecular functions related to proteolysis and protein binding (MF) (Figure 2B). Following identification, the KEGG enrichment analysis indicated that the proteins were predominantly involved in processes such as transport and catabolism, signal transduction, protein folding, sorting, and degradation. Additionally, they were found to be associated with carbohydrate metabolic pathways and functions of the immune and endocrine systems (Figure 2C). Proteins are composed of domains, which are the basic units of their structure, function, and evolution. IPR analysis showed that the protein domains identified were particularly prominent in the following regions: the small GTPase superfamily, serine proteases, the trypsin domain, and the EF-hand domain (Figure 2D).

### 3.3. Identification of the DEPs between Cryptorchid and Normal Testis Group

**Figure 3:**
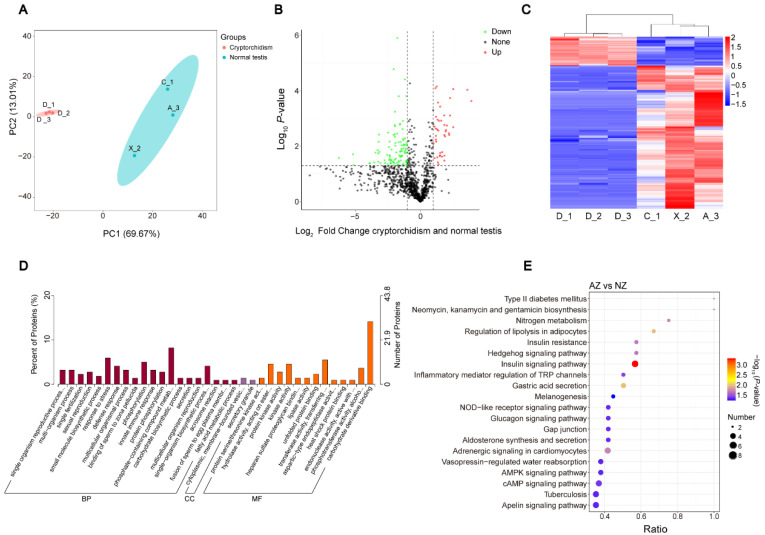
Principal component analysis (PCA) and GO terms and KEGG pathway enrichment DEPs analysis of seminal plasma proteins in cryptorchid and normal testis semen samples. (**A**) PCA of cryptorchid and normal testis semen sample. According to the distribution of PC1 and PC2, the normal testes group were divided into ellipses. PC1. (**B**) Volcano diagram of DEPs between cryptorchid and normal testis group. (**C**) Hierarchical clustering of DEPs. The vertical clustering is the sample clustering, and the horizontal clustering is the protein clustering. Enriched GO terms (**D**), top 20 enriched KEGG pathways (**E**), enrichment of DEPs.

In the present study, PCA score plots demonstrate that the samples from the cryptorchid and normal testes were closely clustered and exhibited a significant degree of distinction. A total of 314 differentially expressed proteins (DEPs) were identified between the two groups (Figure 3B). From the hierarchical clustering map, it is evident that these DEPs are highly effective in distinguishing between the two groups (Figure 3C). In the comparison between cryptorchid and normal testes, we annotated them into three main categories in the GO database (Supplementary Table S2); most of the DEPs were enriched in the below biological process, the response to stress, phosphorylation, the phosphate-containing compound metabolic process, and the single-organism bio-synthetic process. The enriched molecular function terms are hydrolase activity, acting on ester bonds, kinase activity, transferase activity, transferring phosphorus-containing groups, phosphotransferase activity, using the alcohol group as an acceptor, and carbohydrate derivative binding (*p*-value ≤ 0.05) (Figure 3D). Furthermore, of the top 20 enriched KEGG pathways (Figure 3E), the most enriched were the insulin signaling pathway, the regulation of lipolysis in adipocytes, and gastric acid secretion (Supplementary Table S3).

### 3.4. Comprehensive Functional Analysis of DEPs

**Figure 4:**
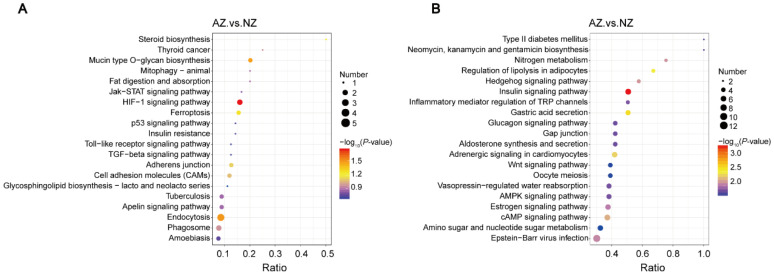
KEGG pathway enriches upregulated and downregulated DEPs between the cryptorchid and the normal testis group. (**A**) The first 20 enriched upregulated KEGG pathways. (**B**) The first 20 enriched downregulated KEGG pathways. The Abscissa in the picture is the ratio of the number of differential proteins in the corresponding pathway to the total number of proteins identified by the pathway. The higher the ratio, the higher the degree of enrichment of differential proteins in this pathway.

Among the DEPS, 53 were upregulated, and the top 10 upregulated genes were D2H6M5, G1L8W6, D2I4F1, G1LC07, G1M5W5, D2GV24, G1LBB8, G1LRS4, G1LVH6, and D2HNJ0. Furthermore, 261 DEPs were downregulated, and the top 10 downregulated genes were D2HS94, G1LY51, D2HDG5, G1MBY6, D2HVQ7, G1LN49, G1LDV3, D2HRQ7, G1L3B7, and G1LX08 (Supplementary Table S4). We carried out KEGG enrichment analysis on differential proteins to obtain more detailed information. The top 20 enriched up- and downregulated protein pathways are shown in Figure 4A and Figure 4B, respectively. In the cryptorchid and normal testis group, the most enriched KEGG pathways were the HIF-1 signaling pathway, mucin-type O-glycan biosynthesis, endocytosis, insulin signaling pathway, gastric acid secretion, and adrenergic signaling in cardiomyocytes pathways.

### 3.5. Network Analysis of the DEPs Involved in Regulating the Semen Quality

**Figure 5:**
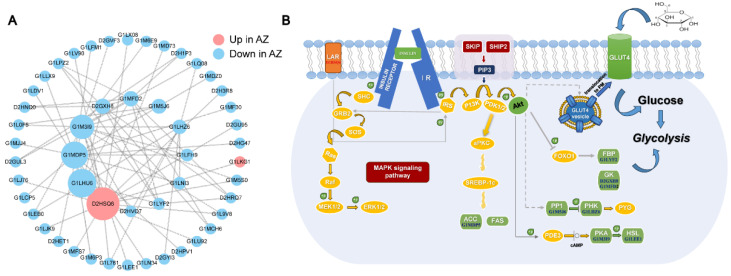
Network analyses of DEPs between the cryptorchid and normal testis groups. (**A**) PPI enrichment analysis showing physical interactions formed among DEPs in the cryptorchid and normal testis groups. (**B**) Insulin signal pathway map involving cryptorchidism sexual characteristics related to seminal plasma proteins.

To further identify the potential key proteins regulating semen quality, we employed the StringDB protein interaction database to map out the interactions among proteins. We then integrated the differentially expressed proteins (DEPs) from both the cryptorchid and the normal testis groups to form a protein–protein interaction (PPI) network (Figure 5A). The top highest degree proteins included D2HSQ6 (protein tyrosine phosphatase, PTPs), G1LHU6 (Pyruvate kinase), G1MDP5 (Acetyl-CoA carboxylase), and G1MFD2 (hexokinase) (Figure 5A). Of these, only D2HSQ6 were highly expressed in the crytorchid group. In addition, we mapped the related proteins to the insulin signal pathway (Figure 5B). PTPs are enzymes that are involved in the deactivation of the Mitogen-Activated Protein Kinase (MAPK) signaling pathway [51], which are crucial for MAPK activation. By dephosphorylating these kinases, protein tyrosine phosphatases serve as key regulators of the MAPK pathway, influencing various cellular responses such as growth, differentiation, and stress reactions.

## 4. Discussion

Cryptorchidism is one of the most common causes of nonobstructive azoospermia (NOA) in adulthood. Clinical data from humans have demonstrated that approximately 20% of azoospermia or infertility male reports involve a history of cryptorchidism. The cause of cryptorchidism is thought to be multi-factorial, including endocrine, environmental, genetic, anatomical, and mechanical factors, so it is usually a relatively complex disease. The natural decline of the testicles after birth forms the basis of the male reproductive system and fertility. However, cryptorchidism is the most common congenital abnormality in male newborns. Long-term testicular ectopia can lead to secondary testicular degeneration and subsequently, to a certain extent, an increase in the risk of testicular malignancy and infertility.

Testicular descent occurs in two phases [52]. During the first phase, before midgestation, testes remain anchored to the inguinal area by the insulin-like hormone 3 (INSL3)-driven development of the gubernaculum [53,54,55]. The second inguinoscrotal phase is dependent on testicular androgens and is usually completed by the time of birth. Mutations of specific genes have rarely been reported in cryptorchidism [56]. However, several risk factors for cryptorchidism, such as preterm birth and low birth weight, have been described. Environmental factors may also have a role in the etiology of cryptorchidism. Future studies on the gene–environment interaction will give new insights to the pathogenesis of cryptorchidism.

As an endangered species that relies on natural conservation, giant pandas have been facing many challenges in ex situ conservation, one of which is the poor natural mating ability of captive male pandas, and male pandas with cryptorchidism have become the focus of attention in this field. To better understand male giant pandas’ reproductive system, we conducted the present study on the semen of both normal and cryptorchid individuals. The main feature was the application of a label-free peptide marker coupled with an LC-MS/MS approach to determine significant differences in semen plasma protein parameters between cryptorchid and normal giant panda testes. Our findings suggest that the sexual characteristics of the cryptorchid individual and his semen quality were closely related to seminal plasma protein.

In the present study, 2059 proteins were identified in the seminal plasma between the cryptorchid and normal testis groups based on proteomic analysis. Of these, we functionally annotated 314 proteins, which were mainly localized in the extracellular region (24.62%), cytoplasm (17.81%), and plasma membrane (15.09%), indicating that their main functions were in the above cell regions. Furthermore, 361 DEPs were identified in the seminal plasma between the cryptorchid and normal testis groups. The data demonstrate that the seminal plasma proteins are crucial in influencing the sperm quality of the giant panda species.

To further elucidate the biological significance of the differentially expressed proteins (DEPs) identified here, functional analyses were conducted. Our results show that the DEPs between the cryptorchid and normal testes were mainly enriched in the GO terms related to response to stress (BP), phosphorylation (BP), the phosphate-containing compound metabolic process (BP), the single-organism biosynthetic process (BP), hydrolase activity, acting on ester bonds (MF), kinase activity, transferase activity, transferring phosphorus-containing groups (MF), phosphotransferase activity, the alcohol group as an acceptor (MF), carbohydrate derivative binding (MF), and cytoplasmic membrane-bounded vesicles (CC). Comparable results have been documented in various species, including humans [57], pigs [58], and chickens [31]. In addition, the DEPs in seminal plasma in both cryptorchid and normal testes were mainly enriched in the KEGG pathways of the regulation of lipolysis in adipocytes, the insulin signaling pathway, and gastric acid secretion.

Furthermore, analyzing the upregulated KEGG enrichment pathway showed that the hif-1/bnip3 pathway, which is evolutionarily conserved, stimulates the processes of mitophagy and mitochondrial fission in the testes of crustaceans when subjected to hypoxic conditions. The hypoxia-inducible factor 1 (HIF-1) signaling cascade is a primitive and robustly conserved pathway that is involved in the hypoxic responses of a vast array of metazoans. Zhao et al. (2023) explored the role of HIF-1 in regulating mitochondrial autophagy in crustacean testes under hypoxia, elucidated the mechanism of HIF-1/bnip3-mediated mitochondrial division and mitochondrial autophagy, and proved that this pathway protects crustaceans from ROS production and apoptosis induced by acute hypoxia [59]. In addition, the health of the testes can be affected by the HIF-1α signaling pathway. It was demonstrated by Wu et al. (2022) that pre-pubertal di-(2-ethylhexyl) phthalate (DEHP, which is a widely used plasticizer) exposure leads to ferroptosis in mouse testes via the HIF-1α/HO-1 signaling pathway [60].

Over the past few years, many studies have observed the behaviors of proteins under this transduction pathway, with the discovery that mouse sperm contain hexokinase type 1 (HK1) with phosphotyrosine residues. Over the past few years, many studies have observed the behaviors of proteins under this transduction pathway, with the discovery that mouse sperm contain hexokinase type 1 (HK1) with phosphotyrosine residues, which is linked to the plasma membrane component of these sperm cells. At least one isoform of hexokinase-1 (HK1) is found in mouse sperm, has an extracellular domain, and behaves as an integral membrane protein [61]. Recent studies have shown that changes in protein tyrosine phosphorylation are associated with sperm motility activation, overactivation, capacitation, and fertilization. Wysocki et al. (2003) purified protein tyrosine phosphatase (PTPase, 500-fold) with acid phosphatase activity from boar seminal vesicle fluid [62]. It was confirmed by Tomes et al. (2003) that both tyrosine kinase and phosphatase play a central role in the extracellular activity of spermatozoa [63]. Calcium transport in sperm plays a significant role in fertilization, particularly in the acrosome reaction. The seminal plasma of the buffalo (Bubalus bubalis) has been found to contain a calmodulin-like protein (CLP), which has undergone preliminary characterization [64]. It has been shown that pyruvate kinase regulation is involved in the metabolic control mechanism of the animal prostate and the seminal vesicle, which affects sperm quality to a certain extent [65,66,67,68,69].

When we tried to explain how these DEPs regulate the semen quality of giant pandas, the results of network analysis showed that the insulin signaling pathway may play a key role in determining the semen quality of giant pandas. In this study, the differentially expressed proteins (DEPs) found in the seminal plasma of giant pandas with cryptorchidism, compared to those with normal testes, showed a marked enrichment in the insulin signaling pathway. It is now evident that the insulin signaling pathway plays a crucial, adaptable, and multifaceted role, evolving into multiple anabolic functions besides glucose homeostasis. Yildirim et al. (2023) showed that the insulin signaling pathway is essential to testicular development, the preservation of microcirculatory function, and the process of spermatogenesis [70]. It regulates vital components of an organism’s health, including growth, lifespan, metabolic processes, and reproductive functions. Part of this diversification has been achieved through the duplications and divergence of both ligands and receptors riding on a common general signal transduction system. A strikingly distinct aspect is its application in the realm of reproduction, notably in the male’s development and the species’ fertility [71]. The DEPs enriched by this pathway include D2GXH8 (hexokinase fragment) D2HSQ6, G1LH26 (Calmodulin 2), G1LYF2 (Fructose-bisphosphatase), G1MDP5 (Acetyl-CoA carboxylase), G1LEE1 (Hormone-sensitive lipase), G1M5J6 (Serine/threonine protein phosphatase), G1M3I9 (cAMP-dependent protein kinase), and G1MFD2 (hexokinase). Among them, D2HSQ6 was significantly upregulated in the seminal plasma of the cryptorchid group, indicating that the protein-related function was promoted in cryptorchidism. Earlier studies have shown that an excess of LAR (leukocyte antigen-related) protein tyrosine phosphatase in muscle tissue leads to a state of insulin resistance [72]. It is plausible that hormonal imbalances or metabolic disturbances associated with cryptorchidism could potentially influence the development of insulin resistance. For instance, cryptorchidism is known to affect testicular function and hormonal regulation, which could hypothetically contribute to metabolic issues [73].

## 5. Conclusions

In this study, we constructed a giant panda proteome map of the cryptorchid seminal plasma group and normal seminal plasma group. The number of proteins identified was 2059, including 314 DEPs, 53 upregulated proteins, and 261 downregulated proteins. Furthermore, bioinformatics analysis showed that these identified DEPs played an important role in insulin signaling, affecting the defects of cryptorchidism and semen quality. A thorough characterization of the proteins in the seminal plasma of the giant panda facilitates a complete understanding of the sperm biology of this endangered species, which is crucial for deciphering the impact of seminal plasma on the fertility of giant pandas. The application of comparative proteomics and the examination of functional networks for these proteins together deepen our systemic comprehension of the proteome within giant panda sperm, offering critical leads in the quest to discern the indispensable, conserved functions of these proteins.

## Data Availability

The mass spectrometry proteomics data have been deposited to the ProteomeXchange Consortium (https://proteomecentral.proteomexchange.org, accessed on 27 March 2024) via the iProX partner repository [74,75] with the dataset identifier PXD051040.

## Supplementary Materials

The following supporting information can be downloaded at: https://www.mdpi.com/article/10.3390/genes15101288/s1, Table S1: GO, COG, KEGG, IPR comment results; Table S2: GO enrichment of differential proteins in seminal plasma proteins of the cryptorchid group and the normal testis group; Table S3: KEGG enrichment of differential proteins in seminal plasma proteins of the cryptorchid group and the normal testis group; Table S4: Analysis list of differential protein results. (When FC ≥ 2.0 and *p*-value ≤ 0.05, up-regulated proteins were screened. When FC ≤ 0.5 and *p*-value ≤ 0.05, down-regulated proteins were screened).
